# Embryonic FAP^+^ lymphoid tissue organizer cells generate the reticular network of adult lymph nodes

**DOI:** 10.1084/jem.20181705

**Published:** 2019-07-19

**Authors:** Alice E. Denton, Edward J. Carr, Lukasz P. Magiera, Andrew J.B. Watts, Douglas T. Fearon

**Affiliations:** 1 Lymphocyte Signaling and Development, Babraham Institute, Cambridge, UK; 2 Department of Medicine, University of Cambridge, Cambridge, UK; 3 Cancer Research UK Cambridge Institute, University of Cambridge, Cambridge, UK; 4 Weill Cornell Medicine and Cold Spring Harbor Laboratory, Cold Spring Harbor, NY

## Abstract

The induction of adaptive immunity is dependent on the structural organization of LNs, which is in turn governed by the stromal cells that underpin LN architecture. Using a novel fate-mapping mouse model, we trace the developmental origin of mesenchymal LN stromal cells (mLNSCs) to a previously undescribed embryonic fibroblast activation protein-α (FAP)^+^ progenitor. FAP^+^ cells of the LN anlagen express lymphotoxin β receptor (LTβR) and vascular cell adhesion molecule (VCAM), but not intercellular adhesion molecule (ICAM), suggesting they are early mesenchymal lymphoid tissue organizer (mLTo) cells. Clonal labeling shows that FAP^+^ progenitors locally differentiate into mLNSCs. This process is also coopted in nonlymphoid tissues in response to infection to facilitate the development of tertiary lymphoid structures, thereby mimicking the process of LN ontogeny in response to infection.

## Abstract (precis)

Denton et al. show that stromal cells of the adult LN derive from a common embryonic FAP^+^ progenitor present when the LN first forms. FAP^+^ progenitors differentiate locally to form the LN stromal network and also become tertiary lymphoid structure stromal cells in nonlymphoid tissues.

## Graphical Abstract

**Figure:**
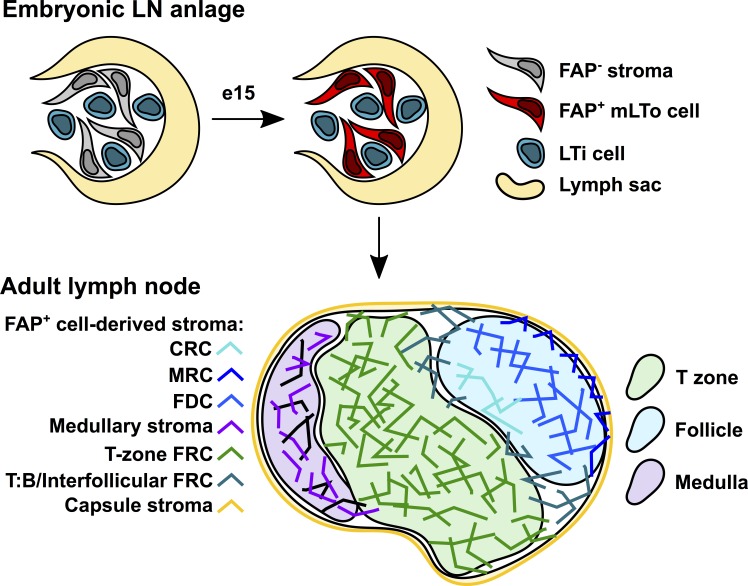

## Introduction

Adaptive immune responses are initiated in secondary lymphoid organs, which contain specialized compartments defined by stromal cells that facilitate interactions between immune cells. While endothelial cells (ECs) mediate lymphocyte ingress and egress, mesenchymal LN stromal cells (mLNSCs) create migration gradients that direct immune cell movement (Cyster, 2005; Denton and Linterman, 2017). Four major subsets of mLNSCs have been described: fibroblastic reticular cells (FRCs) populate the T cell zone, medulla, and interfollicular zone and control T cell localization (Bajénoff et al., 2006); follicular dendritic cells (FDCs) control B cell localization and underpin germinal center (GC) responses (Ansel et al., 2000; Wang et al., 2011); marginal reticular cells (MRCs) play a role in antigen transport (Katakai et al., 2008) and can differentiate into FDCs (Jarjour et al., 2014); and CXCL12-abundant reticular cells (CRCs) form a migratory nexus within the GC (Bannard et al., 2013). Additional subsets of mLNSCs have been recently described (Rodda et al., 2018), suggesting further specialization of mLNSCs. In the absence of functional mLNSCs, adaptive immune responses are compromised (Link et al., 2007; Cremasco et al., 2014; Denton et al., 2014), demonstrating the central role these cells have in immunity.

Despite the importance of mLNSCs, little is known about their origin and differentiation. LN formation is initiated by branching of lymphatic ECs (LECs) and formation of a lymph sac (Srinivasan et al., 2007). Lymphoid tissue inducer cells infiltrate the LN anlagen, and signaling between ECs and lymphoid tissue inducer cells starts LN formation (Onder et al., 2017). Concurrently, mesenchymal precursors seed the anlagen, are primed, and differentiate into mesenchymal lymphoid tissue organizer (mLTo) cells (Bénézech et al., 2010). The relationship between mLTo cells and mLNSCs of the adult LN is poorly understood. While previous studies have identified the origin of splenic stromal cells (Castagnaro et al., 2013), these cells do not become mLNSCs; thus, their development has yet to be fully characterized. While all mLNSCs have a history of CCL19 or CXCL13 expression (Chai et al., 2013; Onder et al., 2017), suggesting that MRCs, FDCs, CRCs, and FRCs have a shared history, there is no clear evidence of whether different LN stromal cell types arise from a single progenitor or whether this happens during development. This is due, in part, to the lack of a system enabling clonal or developmental stage-specific marking.

In this study, we use a novel mouse model to fate-map mLNSCs during embryonic development. We show that LN FRCs, FDCs, and MRCs arise from a novel fibroblast activation protein-α (FAP)-expressing mLTo cell, established by embryonic day (e)15.5 in the inguinal LN (iLN) anlagen. The differentiation of mLNSC types is a local event, and embryonic progenitors of the LN anlage have potential to become FRCs, FDCs, and MRCs. Moreover, FAP^+^ cells in nonlymphoid tissue can be induced to form a stromal cell scaffold that supports the formation of lymphocytic aggregates during infection.

## Results and discussion

### Lineage-tracing FAP-expressing cells in vivo

**Figure 1.**
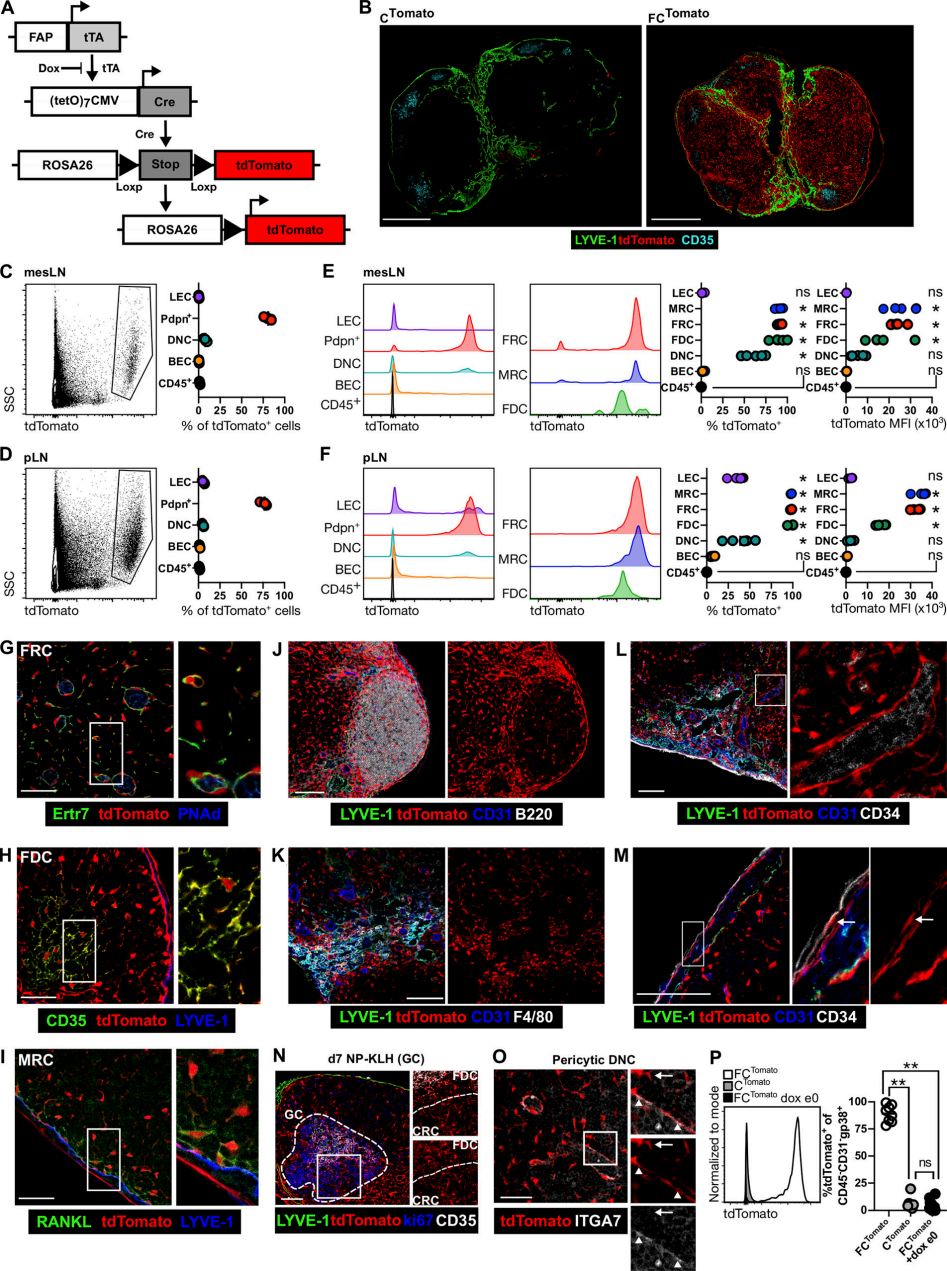
**Adult mLNSCs have a history of FAP expression. (A)** Schematic of FC^Tomato^ mice. **(B)** tdTomato expression in FC^Tomato^ and C^Tomato^ iLN sections. **(C–F)** tdTomato expression by cells from mesLNs (C and E) or pLNs (D and F) from FC^Tomato^ mice. **(C and D)** Phenotype of tdTomato^+^ cells. SSC, side scatter. **(E and F)** tdTomato expression and tdTomato mean fluorescence intensity (MFI) in LN cells. Data represent three independent experiments with four to six mice. Statistical significance was determined by one-way ANOVA, compared with the CD45^+^ group, *, P < 0.001; ns, not significant. **(G–O)** tdTomato^+^ mLNSCs were identified in iLN sections of FC^Tomato^ mice: FRCs (G), FDCs (H), MRCs (I), interfollicular and T:B border mLNSCs (J), medullary mLNSCs (K), CD34^+^ adventitial cells (L), CD34^+^ capsular mLNSCs (M), CRCs in mice immunized 14 d before (N), and perivascular DNCs (O). In O, arrow denotes ITGA7^−^tdTomato^+^ FRC; arrowheads denote ITGA7^+^tdTomato^+^ perivascular cells. **(P)** tdTomato expression in mLNSCs from FC^Tomato^ mice, C^Tomato^ mice, and FC^Tomato^ (+dox e0) mice. Data are compiled from three experiments comprising three to four mice per group. Statistical significance was determined by one-way ANOVA comparing all groups to each other, **, P < 0.0001; ns, not significant. Scale bars, 500 µm (B); 100 µm (J–N); 50 µm (G–I and O). Images represent more than five mice.

We previously identified FAP as a marker of FRCs, but not FDCs (Denton et al., 2014), leading us to hypothesize that FAP expression may provide an approach to probe mLNSC development in vivo. We developed a mouse model to trace cellular lineage based on *Fap* expression. We generated a bacterial artificial chromosome (BAC), inserting the tetracycline transactivator (tTA) at the start ATG of *Fap*, creating Tg(*Fap*^tTA^) mice; this strategy faithfully recapitulates endogenous *Fap* expression (Kraman et al., 2010; Roberts et al., 2013). Breeding Tg(*Fap*^tTA^) mice with Tg(*Teto*^Cre^) and *Rosa26*^lox-stop-lox-tdTomato^ mice to create FC^Tomato^ mice allows fate-mapping of FAP-expressing cells by the induction of indelible tdTomato expression, which can be inhibited by administration of doxycycline (dox; Fig. 1 A). As dox crosses the placenta, administration of dox to mothers during pregnancy permits mapping of FAP^+^ cell fate from different stages of embryogenesis.

TdTomato^+^ cells were located throughout the LN in FC^Tomato^ mice, while few were observed in C^Tomato^ controls that lack the *Fap*^tTA^ transgene (Fig. 1 B). To define the cell types with a history of FAP expression, cells were isolated from mesenteric LNs (mesLNs) and peripheral LNs (pLNs) of untreated FC^Tomato^ mice, and tdTomato^+^ cells were determined. 75–80% of tdTomato^+^ cells were podoplanin (Pdpn)^+^CD31^−^CD45^−^ (Fig. 1, C and D), consistent with mLNSC identity. In mesLNs, >85% of mLNSCs and 45–65% of double-negative cells (DNCs, CD31^−^Pdpn^−^) were tdTomato^+^, with minor LEC or blood EC labeling (Fig. 1 E). tdTomato expression was greater in mLNSCs and DNCs, and the level of tdTomato in ECs was equivalent to that in CD45^+^ cells (Fig. 1 E). In pLNs, >95% of mLNSCs, 30–40% of DNCs, and 25–40% of LECs were tdTomato^+^, while few blood ECs were tdTomato^+^; the mean fluorescence intensity of tdTomato was highest in mLNSCs (Fig. 1 F). Subdivision of the mLNSC fraction into MRCs, FRCs, and FDCs demonstrated that these populations were all tdTomato^+^ and had higher tdTomato expression (Fig. 1, E and F).

Visualization of FRCs, FDCs, and MRCs, as defined by Ertr7, CD35, and receptor activator of nuclear factor-κB ligand (RANKL) expression, respectively, confirmed their history of FAP expression (Fig. 1, G–I). Other mLNSC subsets were also derived from FAP^+^ cells, including CCL19^lo^ and CXCL9^hi^ T zone reticular cells (Fig. 1 J), indolethylamine *N*-methyltransferase–positive stromal cells (Fig. 1 K), and CD34^+^ capsular mLNSCs (Fig. 1 M). CD34^+^ adventitial cells did not have a history of FAP expression (Fig. 1 L), suggesting these cells derive from a different lineage. The GC stromal cells, CRCs, also have a history of FAP expression (Fig. 1 N). The tdTomato^+^ DNCs (Fig. 1, E and F) are likely integrin α7 (ITGA7)^+^ pericytes (Malhotra et al., 2012), as tdTomato expression colocalizes with ITGA7 surrounding high endothelial venules (Fig. 1 O). Previously, we described FAP^+^ macrophages in tumors (Arnold et al., 2014); however, we found no tdTomato expression in LN hematopoietic cells (Fig. S1 A). FAP expression does not mark splenic MRCs or FDCs, although perivascular cells and a small proportion of FRCs are labeled (Fig. S1 B), and both Peyer’s patch (Fig. S1 C) and mesLN (Fig. 1, C and E; and Fig. S1 D) mLNSCs have a history of FAP expression. Our transgenic system is faithful: >85% of mLNSCs isolated from FC^Tomato^ mice were tdTomato^+^, while 4–7% of cells were labeled in C^Tomato^ mice or FC^Tomato^ given dox antecedent to conception (+dox e0; Fig. 1 P). Finally, in line with our previous work (Denton et al., 2014), active FAP expression is detected only in FRCs, as MRCs and FDCs are not tdTomato^+^ when FAP labeling is inhibited in utero (Fig. S1 E). These data demonstrate that a history of FAP expression is a feature of mLNSCs and pericytic DNCs, and that the FC^Tomato^ model is a tractable approach to determine mLNSC developmental origins.

### Adult mLNSCs derive from a FAP^+^ mLTo cell that arises at e15.5

**Figure 2.**
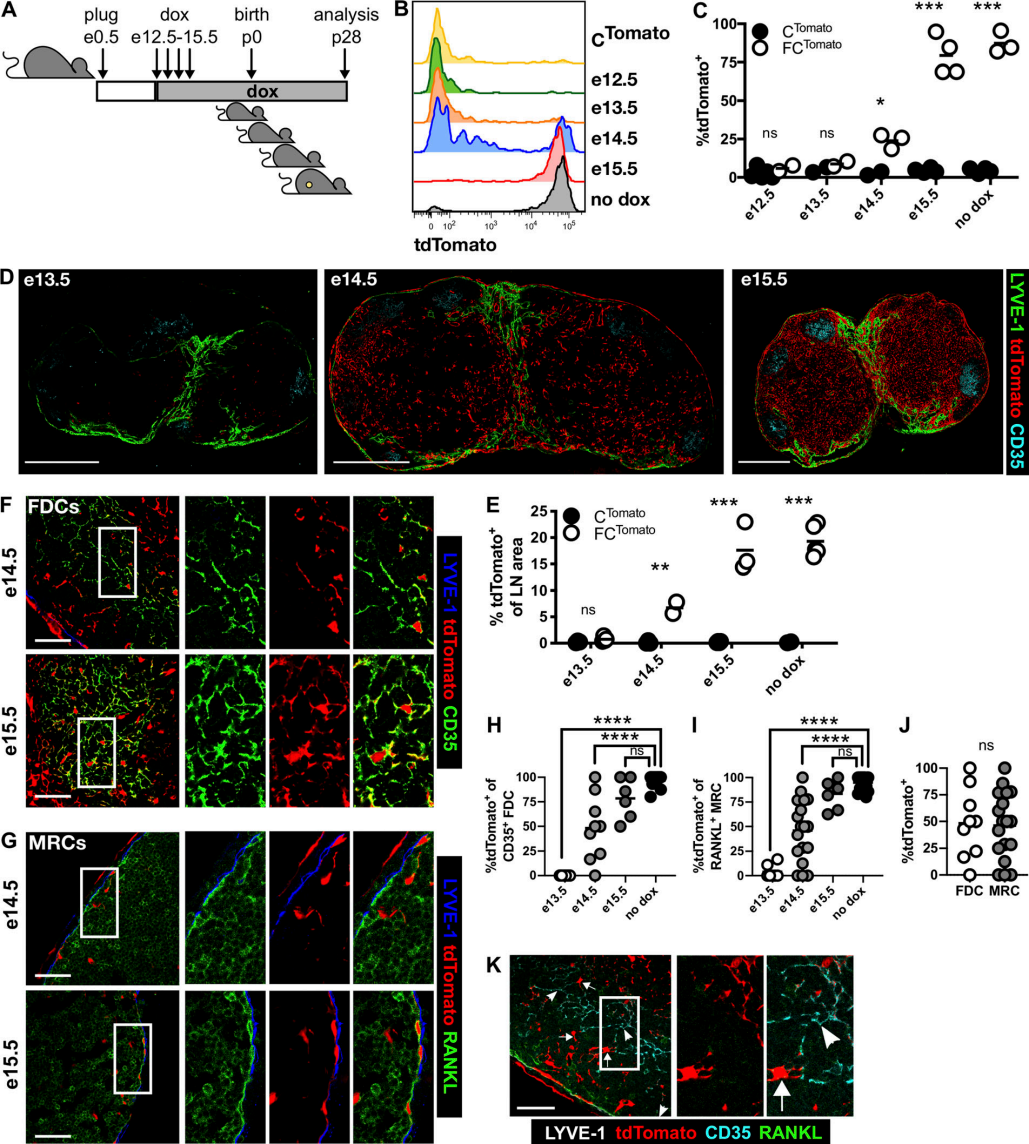
**Adult mLNSCs derive from FAP^+^ cells in the e15.5 iLN anlage. (A)** FC^Tomato^ and littermate C^Tomato^ mice were kept on dox from different stages of embryogenesis until analysis at 4 wk. **(B and C)** The proportion of tdTomato^+^ mLNSCs in iLNs fate-mapped from different stages of embryogenesis. Statistical significance was determined using a two-tailed *t* test, comparing FC^Tomato^ to C^Tomato^ littermates: *, P < 0.05; ***, P < 0.001; ns, not significant. **(D and E)** The LN tdTomato^+^ area was determined in iLNs fate-mapped from different stages of embryogenesis. Scale bars, 500 µm. Images represent two to five individual mice combined from two to three experiments. Statistical significance was determined using a two-tailed *t* test, comparing FC^Tomato^ mice to C^Tomato^ littermates: **, P < 0.01; ***, P < 0.001. **(F and G)** tdTomato^+^ FDCs (F) and MRCs (G) in iLNs fate-mapped from e14.5 and e15.5. Images represent three to five mice. Scale bars, 50 µm. **(H–J)** The proportion of FDCs (H) and MRCs (I) labeled in adult iLNs fate-mapped from embryogenesis. Data represent three or more follicles per mouse, combined from three to five mice in two experiments. Statistical significance was determined by one-way ANOVA, comparing to no dox: ****, P < 0.0001. **(J)** Proportion of FDCs and MRCs that are tdTomato^+^ in FC^Tomato^ (+dox e14.5) mice. Statistical significance was determined using a Mann–Whitney *U* test. **(K)** tdTomato labeling in MRCs and FDCs fate-mapped from e14.5. Arrows and arrowheads indicate tdTomato^+^ and tdTomato^−^ cells, respectively. Scale bar, 50 µm. White boxes indicate enlarged areas. Images represent three to five mice compiled from two experiments.

To define when FAP^+^ cells that become adult mLNSCs first emerge, FC^Tomato^ and littermate C^Tomato^ mice were given dox from different stages of embryogenesis, relative to the detection of vaginal plug (e0.5), and tdTomato expression in mLNSCs was determined in 4-wk-old iLNs (Fig. 2 A). FAP^+^ cells present at e14.5 give rise to 20–25% of mLNSCs, with few mLNSCs labeled before e14.5; all adult mLNSCs derived from cells expressed FAP by e15.5 (Fig. 2, B and C). FAP^+^ cells present at e15.5 gave rise to mLNSCs localized throughout the adult LN, similar to untreated FC^Tomato^ mice (Fig. 2, D and E), while e14.5 FAP^+^ cells did so more sporadically (Fig. 2 D). This suggests that the adult iLN mLNSC ancestor expresses FAP between e14.5 and e15.5; therefore, those labeled at e14.5 reflect the first FAP^+^ progenitors. We used this concept to understand the differentiation of MRCs and FDCs from FAP^+^ progenitors by determining tdTomato labeling of MRCs and FDCs in 4-wk-old iLNs (Fig. 2, F and G). We found that all FDCs and MRCs present in the iLN anlagen derived from a cell that had expressed FAP by e15.5 (Fig. 2, H and I). While the proportion of MRCs and FDCs that had expressed FAP by e14.5 was highly variable, it did not differ between MRCs and FDCs, and labeling was not absolute for a single follicle, as tdTomato^+^ (arrows) and tdTomato^−^ (arrowheads) MRCs and FDCs are closely associated in the same follicle (Fig. 2 J).

**Figure 3.**
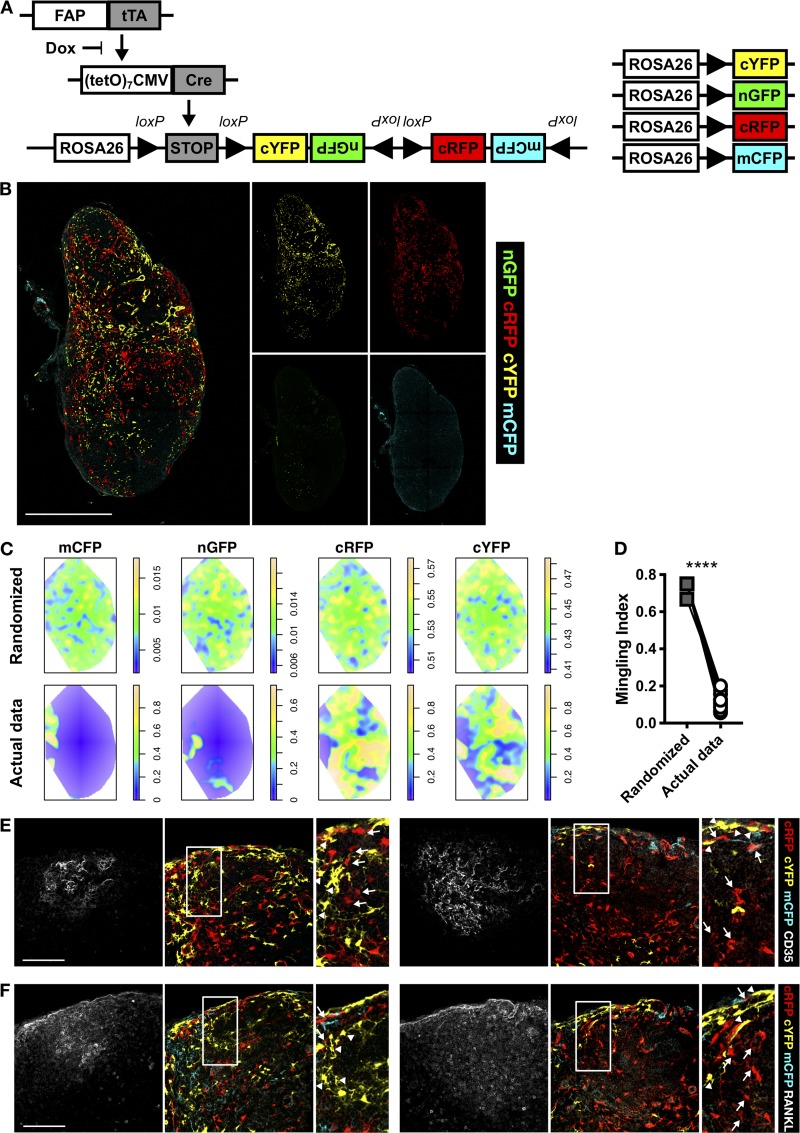
**Adult mLNSC fate is locally determined. (A)** FC^Confetti^ fate-mapping approach. **(B)** Fluorescent proteins mapping FAP expression from e15.5. **(C)** Probability distributions of a randomized Poisson distribution of fluorescent reporters (top), or the actual data (bottom). **(D)** MI was calculated for each iLN and compared with the MI derived from the randomized distributions. Data shown are 15 iLNs combined from two experiments. Statistical significance was determined using a paired *t* test: ****, P < 0.0001. **(E and F)** The relationships of fate-mapped MRCs and FDCs in single follicles was determined in FC^Confetti^ (+dox e15.5) mice. CD35^+^ FDCs (E) and RANKL^+^ MRCs (F), represented as a z-stack encompassing 20 µm. Arrows indicate linked cRFP^+^ cells; arrowheads indicate linked cYFP^+^ cells. See Videos 1 and 2 (E) and Videos 3 and 4 (F). White boxes indicate enlarged areas. Scale bars, 500 µm (B) or 100 µm (E and F). Data represent 15 iLNs from two experiments.

To determine whether differentiation of mLNSCs occurs locally, we bred FC mice with Rosa26^Brainbow2.1^ mice (Snippert et al., 2010) to produce mice in which FAP-expressing cells indelibly express one of nuclear (n)GFP cytoplasmic (c)RFP, cYFP, or membrane (m)CFP from the Rosa locus (FC^Confetti^ mice; Fig. 3 A). Because continual Cre expression drives multiple recombination events, we analyzed iLNs from mice that had been given dox from e15.5 (Fig. 3 B). Sections from FC^Confetti^ (+dox e15.5) iLNs were masked and thresholded to generate spatial maps of fluorescent proteins, which were then converted into probability distributions (Fig. 3 C). To determine whether the distributions were nonrandom, we calculated the mingling index (MI), which determines interspecies mingling within a region of interest (Graz, 2004). Each fluorescently labeled mLNSC was defined as a point, and the nearest four mLNSCs’ colors were determined, measuring species mixing as exclusively one color (MI = 0) or entirely mixed (MI = 1) and averaging this across the whole LN (Fig. 3 C). We found that MI was significantly lower in the data compared with a randomized Poisson distribution of the same data (Fig. 3 D); thus mLNSCs developed in clusters. We compared this analysis to that previously reported for FDC differentiation after immunization (Jarjour et al., 2014), where individual cells are tiled according to Voronoi tessellation (Fig. S2 A). The number of cells forming clusters was significantly higher in the data compared with the randomized distribution (Fig. S2 B). These data are consistent with a single FAP^+^ progenitor locally differentiating into mLNSCs.

Because FDCs do not develop until B cells seed the LN, around birth (van Rees et al., 1985), their lineage history can be mapped using FC^Confetti^ mice administered dox from e15.5. We analyzed FC^Confetti^ fluorescence in MRCs and FDCs in a single follicle to understand MRC–FDC lineage relationships. MRCs and FDCs expressing the same reporter, e.g., cYFP (arrowheads) or cRFP (arrows), were linked (Fig. 3, E and F). Follicles were populated by cells from multiple progenitors (Fig. 2, E and F, left; and Videos 1 and 2) or single progenitors (Fig. 2, E and F, right; and Videos 3 and 4). FDCs were not always linked with MRCs of the same origin (Fig. 2 E). These data support a model in which FDCs may be derived from MRCs (Jarjour et al., 2014), but not all MRCs give rise to FDCs. This may be because of the stochastic nature of FDC differentiation or because a subset of MRCs differentiate into FDCs.

### LN anlagen FAP^+^ cells are perivascular pre-mLTo

**Figure 4.**
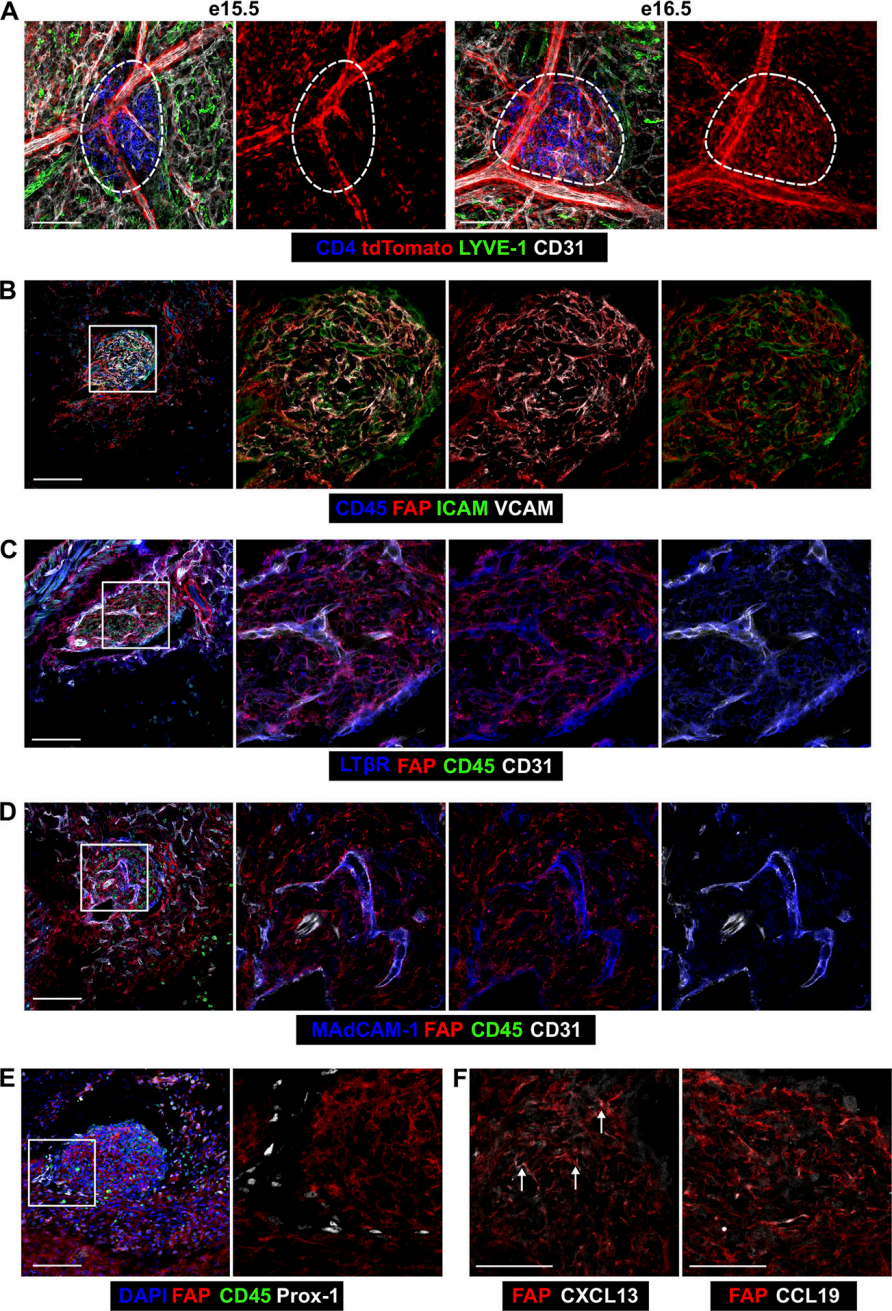
**Adult mLNSCs derive from FAP^+^ pre-mLTo cells. (A)** tdTomato expression in e15.5 and e16.6 FC^Tomato^ iLN anlagen. Scale bars, 100 µm. Dotted line shows the anlage. **(B–F)** FAP^+^ cells in e15.5 iLN anlage were analyzed for mLTo cell markers: ICAM and VCAM (B), LTβR (C), MAdCAM-1 (D), Prox-1 (E), and CXCL13 or CCL19 (F). Scale bars, 100 µm (B–E) or 50 µm (F). White boxes indicate enlarged areas. Arrows in F indicate CXCL13^+^FAP^+^ mLTo cells. Images represent three to six embryos from two to three litters.

To investigate the identity of FAP^+^ LN anlagen cells, we fixed e15.5 and e16.5 FC^Tomato^ embryos and acquired whole-mount images of the iLN anlage, which was defined by CD4 expression (Fig. 4 A). tdTomato^+^ cells lined large blood vessels outside of the anlage and were also in the anlage (Fig. 4 A). Because e15.5 tdTomato^+^ cells give rise to all adult mLNSCs (Fig. 2), we investigated the identity of FAP^+^ cells in the e15.5 iLN anlagen. These cells expressed vascular cell adhesion molecule (VCAM) but not intercellular adhesion molecule (ICAM); ICAM expression was largely restricted to ECs (Fig. 4 B). FAP^+^ cells expressed some lymphotoxin-β receptor (LTβR; Fig. 4 C) but little mucosal addressin cell adhesion molecule 1 (MAdCAM-1; Fig. 4 D); both LTβR and MAdCAM-1 were more strongly expressed by ECs. FAP did not colocalize with Prox-1^+^ cells (Fig. 4 E); thus LECs are not derived from FAP^+^ anlagen cells. Finally, CXCL13 expression was detected in FAP^+^ anlagen cells, but CCL19 was rarely seen (Fig. 4 F). Considering that stromal MAdCAM-1 and ICAM expression are downstream of LTβR signaling in the anlagen (Bénézech et al., 2010), our data suggest that FAP marks an early mLTo cell present before LTβR-mediated priming, which is likely similar to the CXCL13^+^ mLTo cell that seeds the primordial LN anlage (Onder et al., 2017). Our data suggest that FAP^+^ mLTo cells within the nascent LN anlage are a common progenitor for mLNSCs and that the ultimate fate of FAP^+^ mLTo cells is locally determined.

### FAP-derived cells form tertiary lymphoid structure (TLS) stromal cell networks

Because FAP expression is not restricted to lymphoid tissue (Roberts et al., 2013), we investigated whether fibroblasts with a history of FAP expression could give rise to the stromal cell network of TLSs. Influenza A virus (IAV) infection leads to accumulation of T and B cells in the lung and formation of functional TLSs (Moyron-Quiroz et al., 2004; Adachi et al., 2015; Denton et al., 2019). The development of TLSs is associated with remodeling of the local environment, but it is unknown which stromal cells are involved.

**Figure 5.**
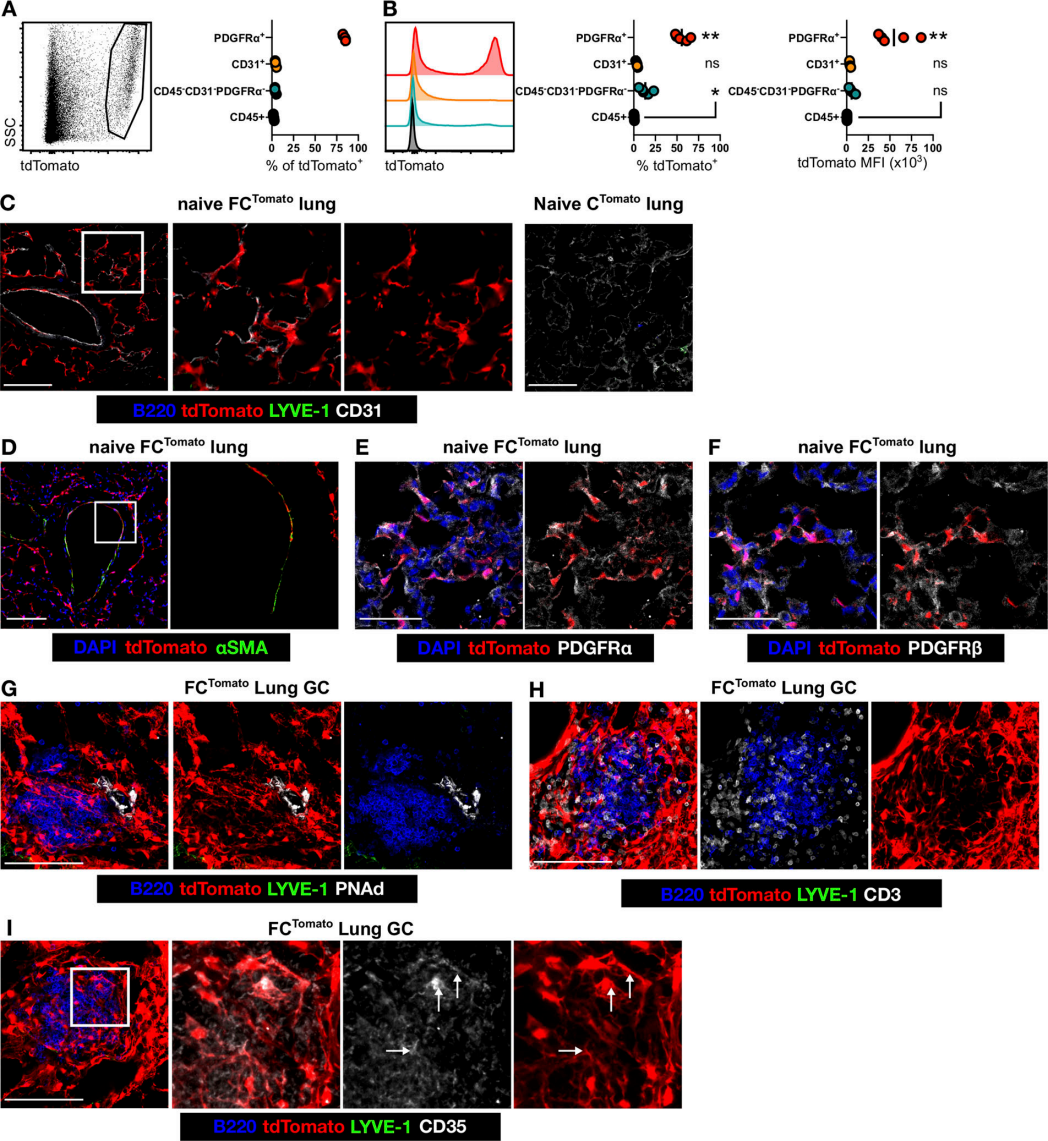
**Virus-induced pulmonary TLSs are supported by a FAP-derived stromal cell network. (A and B)** The proportion of tdTomato^+^ cells expressing PDGFRα, CD31, and/or CD45 (A), the proportion of each population that is tdTomato^+^, and tdTomato mean fluorescence intensity (MFI; B) were determined in naive FC^Tomato^ lungs. Statistical significance was determined by one-way ANOVA, comparing all groups to CD45^+^: *, P < 0.01; **, P < 0.0001; ns, not significant; SSC, side scatter. **(C–F)** tdTomato^+^ cells in naive FC^Tomato^ lung sections showing CD31 (C), αSMA (D), PDGFRα (E), and PDGFRβ (F) expression. The fidelity of transgenic system is demonstrated in C^Tomato^ littermates (C). **(G–I)** TLS development in IAV-infected FC^Tomato^ mice was determined by staining for B cells and the presence of peripheral node addressin^+^ high endothelial venules (G), T cells (H), and CD35^+^ FDC-like cells (I). Arrows in I denote CD35^+^tdTomato^+^ reticular cells. Images in G–I are z-stack projections. Scale bars, 100 µm (C, D, and G–I) or 50 µm (E and F). Data represent at least two independent experiments with three to five mice.

In untreated naive FC^Tomato^ lungs, >75% of tdTomato^+^ cells expressed platelet-derived growth factor receptor-α (PDGFRα) but not CD31 or CD45 (<3%). 10–15% of tdTomato^+^ cells were negative for these markers; these cells may be PDGFRα^−^ fibroblasts or pericytes. While most lung tdTomato^+^ cells were PDGFRα^+^, only 50–60% of lung PDGFRα^+^ cells were tdTomato^+^ (Fig. 5 B), suggesting that lung fibroblasts have mixed origins. FAP-derived lung fibroblasts were distinct from CD31^+^ ECs (Fig. 5 C), closely associated with α-smooth muscle actin (αSMA)^+^ peribronchiolar cells (Fig. 5 D) but not themselves αSMA^+^, and expressed PDGFRα (Fig. 5 E), and occasionally PDGFRβ^+^ (Fig. 5 F). To determine whether the stromal cell network of IAV-induced TLSs is FAP-derived, we infected FC^Tomato^ mice with IAV and determined the development of TLSs 14 d later. TLSs were identified as clusters of B cells and were associated with high endothelial venules (Fig. 5 G) and T cells (Fig. 5 H). We consistently found tdTomato^+^ reticular networks with B cell clusters (Fig. 5, G and H), with an mLNSC-like morphology that is distinct to that of adjacent non-TLS-associated FAP-derived fibroblasts. While CD35^+^ FDC-like cells are rare in IAV-induced TLS (Denton et al., 2019), we found evidence of CD35 expression on FAP-derived TLS reticular networks, although CD35 expression was also found on the B cells (Fig. 5 I). Because of its acute nature, the lymphocytic aggregates and differentiation of reticular stromal cells observed here are less developed than those described in chronic inflammation; however, they are trending toward mature TLS. These data demonstrate that FAP-derived fibroblasts in nonlymphoid tissues can differentiate into mLNSC-like cells, mimicking the developmental process.

### Conclusions

Using a novel fate-mapping approach, we have demonstrated that different mLNSC types share a common embryonic FAP^+^ progenitor. The FAP^+^ pre-mLTo cell arises early during LN development and gives rise to most mLNSC types. FAP-derived fibroblasts can also differentiate into mLNSC-like cells to support TLS formation in adult nonlymphoid tissue. FAP-expressing stromal cells may be a therapeutic target for localized inflammatory diseases in which FAP-expressing cells or ectopic lymphoid structures have been implicated, such as autoimmune disease and cancer.

## Materials and methods

### Mice and treatments

Tg(*Fap*^tTA^) BAC transgenic mice were generated as described (Kraman et al., 2010; Roberts et al., 2013). The tTA construct (PT3073-5; Clontech) was inserted into the *Fap*-containing BAC (RP23-16A15; BACPAC Resources Center) by homologous recombination, and undamaged BAC was purified using a CsCl gradient before pronuclear injection into e0.5 fertilized ova of F1(C57BL/6 × CBA) donors. Tg(*Fap*^tTA^) transgene-positive mice were backcrossed for ≥10 generations to the C57BL/6 background. Tg(*Fap*^tTA^) mice were bred with Tg(*Teto*^Cre^; 1Jaw; Jackson Laboratories) or Tg(*Teto*^Cre^; LC1Bjd; European Mutant Mouse Archive) and *Rosa26*^lox-stop-lox-tdTomato^ or *Rosa26*^Brainbow2.1^ (Confetti) mice (all kind gifts of D. Winton, CRUK Cambridge Institute, Cambridge, UK) to generate FC^Tomato^ or FC^Confetti^ mice. Mice were bred and maintained in specific pathogen–free conditions in the Biological Research Unit at Cancer Research UK (CRUK) Cambridge Institute or the Biological Services Unit at the Babraham Research Campus. For fate-mapping, the stage of embryogenesis was determined relative to plug date, established as e0.5. Dams were administered 1 mg/ml dox (D9891; Sigma-Aldrich) in drinking water with 5% sucrose, and pups were maintained on dox water until analysis at 4 wk of age. For immunization, mice were administered 50 µg 4-hydroxy-3-nitrophenylacetyl hapten–conjugated KLH (Biosearch) emulsified in alum (Thermo Fisher Scientific) subcutaneously under isoflurane anesthesia. For infections, mice were administered 10^4^ pfu IAV (HKx31 strain) intranasally under isoflurane anesthesia. All procedures were approved by the Animal Welfare and Ethical Review Body of the CRUK Cambridge Institute or the Babraham Research Campus and the UK Home Office.

### Flow cytometry

iLNs were enzymatically digested as previously described (Denton et al., 2014), with 0.2 mg/ml collagenase P, 0.1 mg/ml DNase I, and 0.8 mg/ml dispase (all Roche) for 1 h at 37°C to release stromal cells. Single-cell suspensions were blocked (2.4G2 or 93) for 10 min at room temperature then stained with antibodies directed against CD45 (30-F11), CD31 (MEC13.3), CD21/35 (8D9), Pdpn (8.1.1), Ter119, F4/80 (BM8), CD11b (M1/70), B220 (RA3-6B2), CD4 (GK1.5), CD8α (53–6.7), CD11c (N418), MHC II (M5/114.15.2), and/or biotinylated MAdCAM-1 (MECA-367), followed by fluorescently conjugated streptavidin in PBS/2 mM EDTA/2% FCS at 4°C (all BioLegend, BD Biosciences, or eBioscience). Dead cells were excluded using Live/Dead fixable Blue viability dye (Thermo Fisher Scientific) or Fixable Live-Dead e780 (eBioscience). Samples were collected on an LSRFortessa (BD Biosciences) bearing 355-, 405-, 488-, 561-, and 633-nm lasers at the Flow Cytometry facility at the Babraham Research Campus and analyzed using FlowJo (TreeStar).

### Confocal microscopy

iLNs from FC^Tomato^, C^Tomato^, and FC^Confetti^ mice were fixed in 4% paraformaldehyde for 5 h at 4°C and cryoprotected in 20% sucrose overnight before embedding in optimal cutting temperature medium (OCT) and cutting 10–25-µm sections. FC^Tomato^ embryo anlagen were imaged in whole mount: whole embryos were fixed in 4% paraformaldehyde for 5 h at 4°C before removal of the flank skin containing the iLN anlagen. C57BL/6 embryos were fixed in periodate/lysine/paraformaldehyde solution for 5 h at 4°C, cryoprotected in 30% sucrose, and embedded in OCT before serial sectioning at 14 µm. Lungs were inflated with 1:1 4% paraformaldehyde:OCT and fixed in 4% paraformaldehyde for 5 h, cryoprotected, embedded in OCT, and sectioned at 30 µm. Samples were blocked with 1% BSA and 5% goat serum (Sigma-Aldrich), followed by streptavidin/biotin block (Vector Labs) where appropriate, then stained with primary and secondary antibodies. Primary antibodies were anti-Lyve-1 (103-PA50AG; Reliatech), anti-Ertr7 (ab51824; Abcam or T-2109 BMA biomedicals), anti-CD35 (8C12; BD Biosciences), biotinylated anti-CD35 (8C12; BD Biosciences), anti-RANKL (IK22/5; BioLegend), biotinylated antiperipheral node addressin (MECA-79; BioLegend), biotinylated anti-MAdCAM-1 (MECA-367; BioLegend), biotinylated anti-CD31, -594, or -647 (MEC13.3; BioLegend), anti-B220-BV421 or -e660 (RA3-6B2; BioLegend or eBioscience), anti-CD34-APC (RAM34; BD Biosciences), anti-IgD-e450 (11-26c.2a; BioLegend), anti-F4/80 (BM8; BioLegend), anti-CD45-FITC or -e450 (30-F11; BioLegend), anti-CD3-APC (17A2; eBioscience), anti-Prox-1 (AF2727; R&D Systems), anti-VCAM-647 (MVCAM.A; BioLegend), anti-ICAM-FITC (YN1/1.7.4; BioLegend), anti-CXCL13 (AF470; R&D Systems), anti-CCL19 (AF880; R&D Systems), biotinylated anti-LTβR (eBio3C8; eBioscience), anti-FAP (ABT11; Merck), anti-αSMA (1A4; Abcam), anti-PDGFRα (APA5; BioLegend), anti-PDGFRβ (APB5; Invitrogen), and/or CD4-BV421 (RM4-5; BioLegend). Primary antibodies were detected with Alexa Fluor 405–, 488–, or 647–conjugated secondary antibodies and/or streptavidin 405, BV421, 488, or 647 (all BioLegend or Thermo Fisher Scientific). Some images were counterstained with DAPI (Sigma-Aldrich) to identify nuclei. Detection of mouse antibodies on murine tissue was conducted as described (Goodpaster and Randolph-Habecker, 2014). Sections were imaged using a Zeiss LSM880 harboring 405-, 432-, 488-, 561-, and 633-nm lasers, using the Imaging Facility at the Babraham Research Campus. Whole LN images were tiled using Zen Black (Zeiss) software using a 20×/0.50 air objective, and detailed images were captured using 40×/1.40 or 63×/1.40 oil objectives. Image channels were collected separately and analyzed and compiled using Zen Black (Zeiss) or Fiji (National Institutes of Health) software.

The LN area comprising tdTomato^+^ cells was calculated as follows: 20× tiled images of iLNs were manually masked based on Lyve-1 and DAPI expression, and the tdTomato signal was thresholded and converted to a binary signal. The proportion of LN area that was tdTomato^+^ was then determined using Area Fraction in Fiji.

To calculate MI, 20× tiled images from FC^Confetti^ mice were masked using all available colorspace points and thresholded to generate spatial maps of nGFP, cRFP, cYFP, and mCFP expression across the whole LN. The LN boundaries were determined programmatically using an overlay of all four colors. These spatial point patterns were then converted into probability distributions, similar to that already described (Setiadi et al., 2010). The MI was used as described in Graz (2004). Poisson distributions were used to generate null distributions. All analysis was performed in RStudio (running R 3.5.1), using the following packages: EBIImage (tiff import, masking, thresholding; v4.18.3; Pau et al., 2010); spatstat (LN boundary [a convex hull]; probability distributions and randomization; Baddeley and Turner, 2005); spatial segregation (MI; https://CRAN.R-project.org/package=spatialsegregation). These analyses are possible on a standard desktop computer, but the time cost of calculations of MI for a whole iLN (point patterns containing hundreds of thousands of points) meant that we opted to run this component of the image analysis in parallel on a compute cluster (using R 3.5.1, with a fork for each LN).

To determine Voronoi clustering, a point pattern was generated as described for the MI analysis. From this point pattern we generated Voronoi tiles using spatstat library (Baddeley and Turner, 2005), where the “space” between cells is allocated to its nearest cell, such that the tiles tesselate across the whole LN area. Adjacent tiles of the same fluorescent protein were combined into a cluster (tiles were converted to a SpatialPolygon object and unions found using maptools, https://CRAN.R-project.org/package=maptools). The average number of cells per cluster was defined by dividing the number of original tiles by the number of clusters. The original point pattern was downsampled by 1,000-fold for computational practicality. This results in ∼2,000 Voronoi tiles per LN, which merge to a few hundred clusters. Each fluorescent marker was downsampled separately to maintain the mCFP:cYFP:cRFP:nGFP ratio in the analyzed subsample. To generate a reference distribution for our Voronoi analysis, we took each LN’s point pattern in turn and made 10 random (Poisson) null distributions. Each of the 10 iterations was downsampled and processed as above. The number of cells per cluster was calculated for each iteration and averaged using means.

### Online supplemental material

Fig. S1 shows tdTomato expression in LN CD45^+^ cells, spleens, Peyer’s patches, and mesLNs of FC^Tomato^ mice and tdTomato expression in LNs from FC^Tomato^ mice given dox from e0 to postnatal day 0. Fig. S2 shows Voronoi analysis of iLNs from FC^Confetti^ (+dox e15.5) mice. Videos 1, 2, 3, and 4 show zstacks of CD35^+^ FDCs and RANKL^+^ MRCs in FC^Confetti^ (+dox e15.5) mice.

## Supplementary Material

Supplemental Materials (PDF)

Video 1

Video 2

Video 3

Video 4
