# Supplementary material for: Embryonic FAP+ lymphoid tissue organizer cells generate the reticular network of adult lymph nodes
Source: J Exp Med. 2019 Jul 19;216(10):2242–52. doi: 10.1084/jem.20181705 (PMC6780995; doi:10.1084/jem.20181705)
Supplement: Supplemental Materials (PDF) [file JEM_20181705_sm.pdf]

Supplemental material

Denton et al., https://doi.org/10.1084/jem.20181705

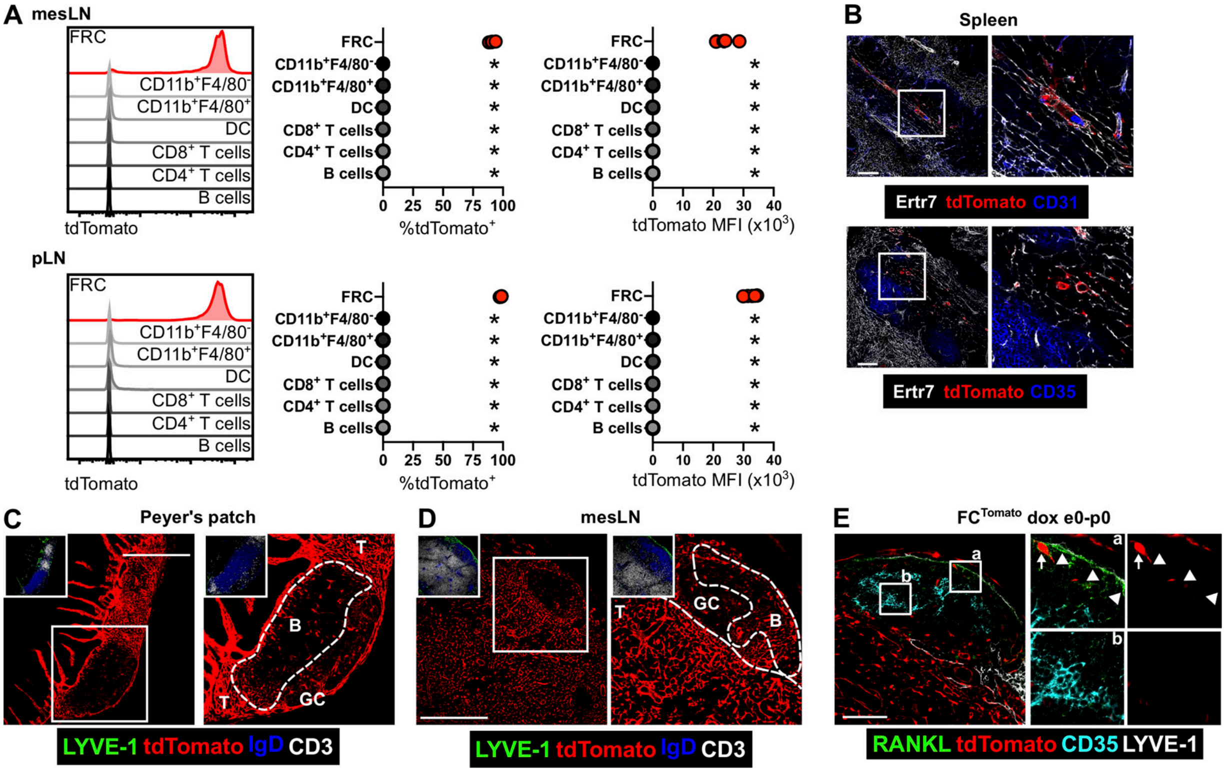

Figure S1. **Characterization of tdTomato⁺ cells in FC^{Tomato} mice. (A)** tdTomato expression among CD45⁺ cells of mesLNs and pLNs, showing FRCs as a positive control for labeling, and CD11b⁺F4/80⁻ and CD11b⁺F4/80⁺ macrophages, CD11c⁺MHC II⁺ DCs, CD8⁺ T cells, and CD4⁺ T cells and B cells. Shown are representative flow cytometry profiles, the proportion of each population that is tdTomato⁺, and the mean fluorescence intensity (MFI) of tdTomato in each population. Statistical significance was determined using a one-way ANOVA, comparing all groups to the FRC group: *, $P < 0.0001$. **(B)** tdTomato⁺ cells in FC^{Tomato} mouse spleens were stained for Ertr7 and CD31 (top) or CD35 (bottom) to identify FRCs alongside blood vessels or FDCs, respectively. **(C and D)** tdTomato⁺ cells were readily identifiable in FC^{Tomato} mouse Peyer's patches (C) and mesLNs (D). LN regions indicated by IgD, CD3, and Lyve-1 staining (shown in inset): B, B cell follicle; T, T cell zone. **(E)** FC^{Tomato} mice were administered dox from before conception until birth, and iLNs were analyzed at 4 wk of age. MRCs (RANKL⁺) are shown in inset a, and FDCs (CD35⁺) are shown in inset b. Lymphatics are identified by Lyve-1, and cells that express FAP after birth are tdTomato⁺. Arrows indicate tdTomato⁺ MRCs; arrowheads indicate tdTomato⁻ MRCs. Scale bars, 500 µm (C and D), 100 µm (B and E). Data are representative of three independent experiments with three to six mice (A–D) or two experiments with two to three mice (F).

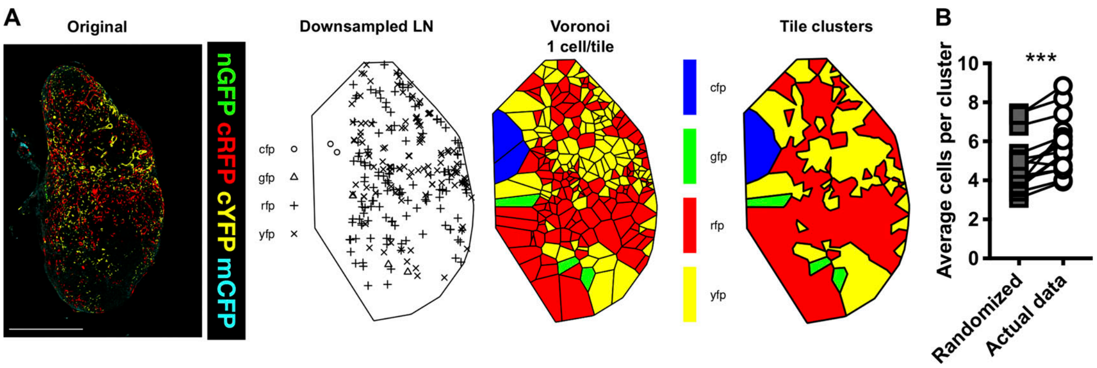

Figure S2. **Voronoi tessellation in FC^{Confetti} mice.** FC^{Confetti} mice were administered dox from e15.5, and the distribution of fluorescent proteins was determined at 4 wk of age in the iLN as in Fig. 3. **(A)** The original data (left) was downsampled by 1,000-fold and converted into Voronoi tessellation tiles representing one cell per tile, as described in Materials and methods. Adjacent tiles of the same color were then simplified into one cluster. Scale bar, 500 µm. **(B)** The average number of cells per cluster was determined, relative to 10 randomized Poisson simulations of the same data, for each iLN. Each point represents an individual iLN; random and actual data pairs are connected by a line. Statistical significance was determined using a paired *t* test: ***, P < 0.001. Data are representative of 15 iLNs compiled from two independent experiments.

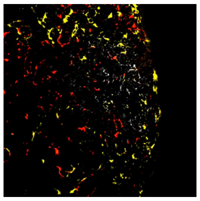

Video 1. **Z-stack movie of FC^{Confetti} (+dox e15.5) and CD35 labeling an individual B cell follicle.** Related to Fig. 3 E, left. FC^{Confetti} mice were administered dox from e15.5, and the distribution of fluorescent proteins was determined at 4 wk of age in the iLN by confocal microscopy as in Fig. 3 E. Video showing a z-stack projection encompassing 20 µm of CD35^{+} FDCs labeled with cRFP, cYFP, or mCFP. Video is 14 frames per second.

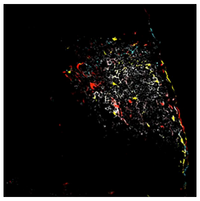

Video 2. **Z-stack movie of FC^{Confetti} (+dox e15.5) and RANKL labeling an individual B cell follicle.** Related to Fig. 3 F, left. FC^{Confetti} mice were administered dox from e15.5, and the distribution of fluorescent proteins was determined at 4 wk of age in the iLN by confocal microscopy as in Fig. 3 F. Video showing a z-stack projection encompassing 20 µm of RANKL^{+} MRCs labeled with cRFP, cYFP, or mCFP. Video is 14 frames per second.

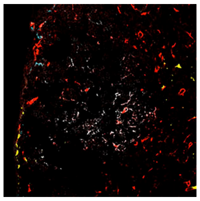

Video 3. **Z-stack movie of FC^{Confetti} (+dox e15.5) and CD35 labeling an individual B cell follicle.** Related to Fig. 3 E, right. FC^{Confetti} mice were administered dox from e15.5, and the distribution of fluorescent proteins was determined at 4 wk of age in the iLN by confocal microscopy as in Fig. 3 E. Video showing a z-stack projection encompassing 20 µm of CD35^{+} FDCs labeled with cRFP, cYFP, or mCFP. Video is 14 frames per second.

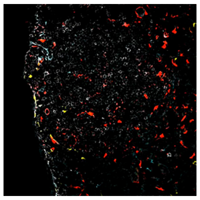

Video 4. **Z-stack movie of FC^{Confetti} (+dox e15.5) and RANKL labeling an individual B cell follicle.** Related to Fig. 3 F, right. FC^{Confetti} mice were administered dox from e15.5, and the distribution of fluorescent proteins was determined at 4 wk of age in the iLN by confocal microscopy as in Fig. 3 F. Video showing a z-stack projection encompassing 20 µm of RANKL^{+} MRCs labeled with cRFP, cYFP, or mCFP. Video is 14 frames per second.
